# Loganin Modulates Inflammatory Signalling and Epidermal Alterations in an Imiquimod‐Induced Psoriasis Mouse Model

**DOI:** 10.1002/jbt.71178

**Published:** 2026-11-10

**Authors:** Eda Acikgoz, Cafer Tas, Mustafa Cakir, Seda Keskin

**Affiliations:** ^1^ Department of Histology and Embryology, Faculty of Medicine Van Yuzuncu Yil University Van Turkey; ^2^ Department of Medical Biology, Faculty of Medicine Van Yuzuncu Yil University Van Turkey

**Keywords:** imiquimod‐induced psoriasis model, inflammatory signalling pathways, Loganin, NLRP3 inflammasome, skin inflammation

## Abstract

Psoriasis is a chronic immune‐mediated inflammatory skin disease driven by abnormal interactions among keratinocytes, immune cells and cytokine networks. Dysregulated inflammatory signalling has been shown to exacerbate psoriatic inflammation. Loganin (LOG), a natural iridoid glycoside, exhibits anti‐inflammatory effects in experimental models, yet its relevance to psoriasis remains poorly characterised. This study investigated the effects of LOG during the development of IMQ‐induced psoriasis‐like inflammation, evaluating its influence on inflammatory signalling and associated tissue responses using PASI scoring, histopathology and stereology. Molecular and cellular alterations were assessed using qPCR, immunohistochemistry, immunofluorescence, and ELISA to determine changes in pro‐inflammatory cytokine profiles, NOD‐like receptor protein 3 (NLRP3) inflammasome components, markers of epidermal proliferation and keratinocyte remodelling, and vascular responses. Topical IMQ induced characteristic psoriatic alterations, including epidermal hyperplasia, immune cell infiltration, microvascular activation, and elevated pro‐inflammatory cytokines. LOG significantly attenuated IMQ‐associated body weight loss, reduced psoriatic clinical severity scores and epidermal thickening, and decreased proliferating cell nuclear antigen (PCNA) and cytokeratin‐17 (CK‐17) immunoreactivity. Furthermore, LOG attenuated vascular endothelial growth factor receptor‐2 (VEGFR2) immunoreactivity and decreased interleukin‐17A (IL‐17A) and interleukin‐1β (IL‐1β) levels, suggesting modulation of inflammatory and vascular responses. LOG also downregulated the expression of NLRP3, apoptosis‐associated speck‐like protein (ASC), and caspase‐1. Collectively, these findings indicate that LOG attenuates IMQ‐induced inflammatory responses and is associated with reduced expression of NLRP3 inflammasome‐related components. These findings support the anti‐inflammatory effects of LOG in the IMQ‐induced psoriasis model while indicating that further functional studies are required to determine whether LOG directly affects NLRP3 inflammasome activation.

## Graphical abstract

BALB/c mice were topically treated with imiquimod (IMQ) to induce psoriatic skin inflammation and co‐administered loganin (LOG) for seven consecutive days. Disease severity was evaluated using PASI scoring, body weight monitoring, skin thickness measurements, histopathological examination and stereological analysis. Molecular and cellular alterations were assessed by qPCR, immunohistochemistry, immunofluorescence, and ELISA. LOG suppressed IMQ‐induced inflammatory responses, as evidenced by reduced expression of pro‐inflammatory cytokines, including IL‐1β and IL‐17A, and downregulation of inflammasome‐associated components (NLRP3, ASC, caspase‐1). In addition, LOG reduced markers of epidermal hyperproliferation and vascular remodelling (PCNA, CK‐17, VEGFR2), contributing to partial restoration of cutaneous structure. Collectively, these findings indicate that LOG modulates inflammatory signalling and associated cellular responses in the IMQ‐induced psoriasis mouse model.

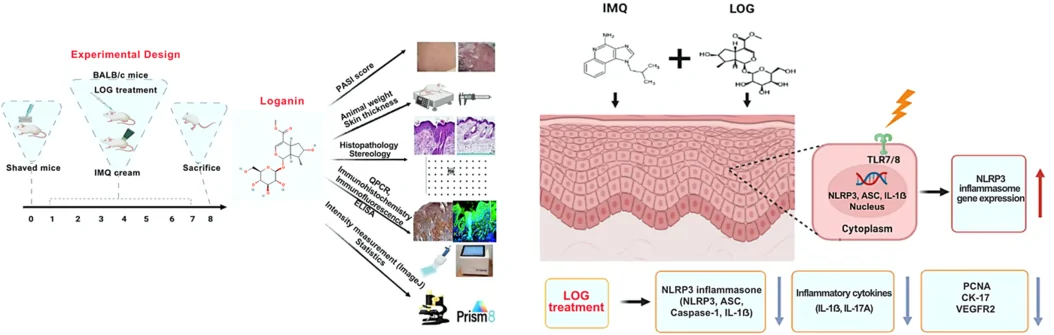

## 1 Introduction

Psoriasis is a chronic, immune‐mediated inflammatory skin disorder affecting more than 125 million people worldwide and increasingly recognised as a systemic condition associated with substantial physical, psychological and socioeconomic burden. Beyond its characteristic cutaneous manifestations, psoriasis is associated with impaired quality of life and multisystem involvement [1]. Its pathogenesis is driven by dysregulated crosstalk between keratinocytes and immune cells, with a prominent role for cytokine networks centred on interleukin‐17A (IL‐17A) and tumour necrosis factor‐α (TNF‐α), which collectively sustain epidermal hyperplasia and chronic inflammation [2].

Accumulating evidence indicates that inflammasome‐associated signalling contributes to the inflammatory network underlying psoriasis, with particular attention to the NOD‐like receptor protein 3 (NLRP3) inflammasome. Increased expression of NLRP3‐associated components has been reported in psoriatic lesions, along with elevated interleukin‐1β (IL‐1β), which can promote Th17 differentiation and IL‐17A production, thereby reinforcing keratinocyte activation and inflammatory persistence [2, 3, 4]. Canonical NLRP3 inflammasome activation involves an initial priming step, commonly associated with NF‐κB‐dependent transcription of NLRP3 and pro‐IL‐1β, followed by inflammasome assembly, caspase‐1 activation, and processing of pro‐inflammatory cytokines. However, NLRP3 dysregulation in psoriasis is increasingly considered part of a broader inflammatory network involving interconnected innate immune, cytokine, and keratinocyte‐associated responses rather than an isolated upstream driver [1, 2, 3, 4]. The imiquimod (IMQ)‐induced psoriasis mouse model provides a well‐established experimental system for investigating these interconnected inflammatory processes. As a toll‐like receptor 7/8 (TLR7/8) agonist, IMQ induces innate immune activation and downstream inflammatory responses, including the IL‐23/IL‐17 axis, resulting in epidermal hyperplasia and immune cell infiltration that reproduce key features of human psoriasis [5]. Accordingly, the IMQ model provides a relevant platform for evaluating bioactive compounds that may modulate psoriasis‐associated inflammatory signalling, including NLRP3 inflammasome‐associated responses.

Loganin (LOG), a naturally occurring iridoid glycoside, has attracted increasing attention for its reported anti‐inflammatory, antidiabetic and neuroprotective properties [6, 7, 8, 9]. Previous studies in non‐psoriatic inflammatory models have reported that LOG can influence the nuclear factor‐κB (NF‐κB) and mitogen‐activated protein kinase (MAPK)‐associated signalling and reduce the expression of NLRP3 inflammasome‐related components [8, 9, 10]. However, whether these signalling mechanisms contribute to the effects of LOG in psoriatic skin inflammation remains unclear. These mechanisms are highly relevant to psoriasis, a disease in which NF‐κB–driven cytokine production, MAPK‐mediated keratinocyte activation and inflammasome‐associated IL‐1β signalling contribute to disease amplification. Despite these indications, the potential impact of LOG on psoriatic inflammation has not been comprehensively examined, and its influence on epidermal hyperplasia, keratinocyte‐associated pathways, or inflammasome‐related components remains largely unexplored.

In this study, we investigated the effects of LOG in the IMQ‐induced psoriasis mouse model using histopathological, immunohistochemical, molecular and biochemical approaches. We assessed clinical severity of psoriasis, alterations in epidermal and dermal density, and markers of keratinocyte proliferation (PCNA, CK‐17), angiogenesis (VEGFR2) and inflammation, including inflammasome components (NLRP3, ASC, caspase‐1, IL‐1β). By integrating these readouts, we aimed to determine whether LOG modulates IMQ‐induced psoriatic inflammation and to provide a mechanistic context for its potential relevance as a bioactive compound in inflammatory skin disease.

## 2 Materials and Methods

### 2.1 Chemicals and Antibodies

Loganin (Ambeed, A106244, Illinois, USA) was dissolved in saline and stored in aliquots at −80°C. Imiquimod cream (Aldara 5%, Meda Pharma) was used as the topical agent. Depilatory cream (Veet, Reckitt Benckiser, France) and Vaseline (Unilever, UK) were obtained from a local pharmacy. ELISA kits for IL‐17A (E1143Mo) and IL‐1β (E0192Mo) were purchased from BT Laboratory Co. Ltd. (China). Primary antibodies included NLRP3 (E‐AB‐65952) and ASC/TMS1 (E‐AB‐63530) from Elabscience (China), IL‐1β (sc‐52012), caspase‐1 (sc‐392736), PCNA (sc‐56) and CK‐17 (sc‐393002) from Santa Cruz Biotechnology (USA), and VEGFR2 (9698S) from Cell Signalling Technology (USA). All other reagents were of analytical grade.

### 2.2 Experimental Design

A total of 35 healthy female BALB/c mice (25–30 g, 4–6 weeks) were obtained from the Van Yuzuncu Yil University Laboratory Animal Centre (Van, Turkey). Mice were housed under standard conditions (22 ± 1°C, 50 ± 5% humidity, 12‐h light/dark cycle) with *ad libitum* access to food and water. All procedures were approved by the Van Yuzuncu Yil University Experimental Ethics Committee (2021/10–14) and conducted in accordance with the ARRIVE guidelines.

### 2.3 Establishment of the IMQ‐Induced Psoriasis Mouse Model

An IMQ‐induced psoriasis mouse model was established as previously described, with minor modifications [5]. Briefly, mice were randomly assigned to five experimental groups (*n* = 7 per group): Control (CONT), Saline + Vaseline (SAL + VAS), Loganin (LOG), IMQ and IMQ + Loganin (IMQ + LOG). Prior to topical IMQ cream application, dorsal skin hair was removed with an electric clipper (Braun Mini Clipper, Wahl, Sterling, IL, USA), followed by depilatory cream (Veet) to ensure complete hair removal. Animals in the CONT group received no further intervention after shaving. The SAL + VAS group served as the vehicle control. Vaseline was applied topically once daily for 7 consecutive days to the shaved dorsal skin (62.5 mg/day) and the inner surface of the right ear (20 mg/cm^2^/day). Subsequently, sterile saline was administered subcutaneously (s.c.) to the dorsal region (100 μL) and the right ear (50 μL) [11, 12]. The experimental model was induced by daily topical application of 5% IMQ cream to the shaved dorsal skin (62.5 mg) and the right ear (3.125 mg) for 7 consecutive days [5]. The LOG group received LOG alone, whereas the IMQ + LOG group received both LOG and IMQ for 7 consecutive days. LOG was administered s.c. to both the dorsal skin region and the right ear. In the dorsal region, LOG was administered at 50 mg/kg/day, with the injection volume limited to 100 μL per injection site [11, 12]. The right ear received LOG at 12.5 mg/kg/day [13] with the injection volume limited to 50 μL per injection site. In the IMQ + LOG group, LOG was administered once daily before each topical IMQ application throughout the 7‐day experimental period. IMQ was subsequently applied topically to the shaved dorsal skin (62.5 mg/day) and the inner surface of the right ear (20 mg/cm^2^/day). Thus, LOG and IMQ were administered to the corresponding dorsal and right ear regions via s.c. and topical routes, respectively. LOG was freshly dissolved in sterile saline before each administration. The dorsal LOG dose of 50 mg/kg/day was selected based on previously published in vivo studies reporting anti‐inflammatory or cytoprotective effects of LOG at this dose without evident toxicity [14, 15]. The present study therefore evaluated the effects of this previously established dose during the induction phase of IMQ‐induced psoriasis‐like inflammation. The subcutaneous route was selected to provide controlled administration of LOG while avoiding potential variability associated with topical formulation and cutaneous penetration. This approach enabled the evaluation of subcutaneously administered LOG during IMQ‐induced skin inflammation; however, topical administration was not assessed in the present study. Dorsal skin thickness and the severity of psoriatic skin lesions were assessed daily by two independent, group‐allocation‐blinded investigators using a digital micrometre and a modified Psoriasis Area and Severity Index (PASI) scoring system (scale 0–4), respectively. Body weight and gross skin morphology were monitored throughout the experimental period to assess general health and tolerability. At the end of the experimental period, mice were anaesthetised and euthanised for sample collection. Blood samples were obtained by cardiac puncture, allowed to clot at room temperature, and then centrifuged at 3000 × *g* for 20 min to obtain serum. Skin samples from the dorsal area were harvested for subsequent histopathological, immunohistochemical, molecular and biochemical analysis.

### 2.4 Histological Evaluation

After sacrifice, dorsal and ear skin tissue samples were fixed in 10% buffered formalin for 48 h, embedded in paraffin, and sectioned at 5 μm using a Leica rotary microtome. Sections were stained with haematoxylin‐eosin and Masson's trichrome with aniline blue (04‐010802, Bio‐Optica, Italy), as previously described [16]. Psoriasis‐related histopathological features were evaluated under a light microscope (BX40, Olympus, Tokyo, Japan) using the Baker scoring system, with a maximum total score of 10 [17]. Histopathological parameters were assigned parameter‐specific weighted scores rather than a uniform ordinal scale. The evaluated criteria included Munro abscesses (2.0), hyperkeratosis (0.5), parakeratosis (1.0), thinning of the epidermis above the dermal papillae (0.5), elongation and clubbing of the rete ridges (1.5), acanthosis (0.5), absence of the granular layer (1.0), dermal lymphocytic infiltration [mild (0.5), moderate (1.0) or marked (2.0)] and papillary congestion (0.5). The individual parameter scores were summed to obtain the total Baker histopathological score (range, 0–10), with higher scores indicating more pronounced psoriasis‐like histopathological alterations.

### 2.5 Histomorphometric Measurements

The effects of LOG on keratinocyte hyperproliferation‐induced epidermal thickening were quantified in H&E‐stained dorsal and ear skin sections using ImageJ. For each mouse, six non‐overlapping images obtained from different tissue regions were analysed at ×40 magnification. Measurements from the six images were averaged to obtain a single animal‐level value prior to statistical analysis. Collagen fibre density was analysed in Masson's trichrome‐stained sections. For each mouse, five non‐overlapping microscopic fields obtained from different tissue regions were evaluated at ×40 magnification. Vascular changes, including vessel diameter and coverage area, were assessed in 10 non‐overlapping regions from different areas of the dorsal skin of each mouse at ×20 magnification using the lasso tool in ImageJ (Fiji) [18]. For all histomorphometric analyses, measurements from multiple images, fields, or regions within the same mouse were averaged to obtain a single animal‐level value prior to statistical analysis. Thus, the individual mouse was considered the biological and experimental unit (*n* = 7 mice/group), whereas individual microscopic images, fields, or regions represented within‐animal subsamples.

### 2.6 Stereological Estimation of Epidermal and Dermal Volume Density

Systematic random sampling was used to assess epidermal and dermal volume ratios using the Cavalieri principle [19]. A random start yielded 12 Section (5 µm thick) from each paraffin block at 60‐section intervals. H&E‐stained sections were analysed stereologically at 4× magnification with a Zeiss Axioskop 40 microscope (Germany) and a Canon EOS 1000D camera (Japan). A point‐counting grid (interpoint distance: 400 µm; *a*/*p* = 16,000 µm^2^) was superimposed on digital photographs. Stereology software (Shtereom 1.5, Denizli, Turkiye) was used to determine the volume densities of the epidermis and dermis with random test grids. Formula for calculating each structure's volume density (*V_V_*):

$V_{V}\;(\mathrm{structure},\;\mathrm{reference})=\Sigma P(\mathrm{structure})/\Sigma P(\mathrm{reference})\times 100$

Σ*P*(structure) is the total number of test points that hit the target structure (epidermis or dermis), and Σ*P*(reference) is the total number of points that hit the reference tissue (epidermis + dermis). The coefficient of error (CE) and the coefficient of variation (CV) were calculated to assess reliability and reproducibility, as described in previous studies [20, 21].

### 2.7 Evaluation of PCNA, CK‐17 and VEGFR2 Expressions by Immunofluorescence (IF)

Tissue sections were prepared from formalin‐fixed, paraffin‐embedded blocks for routine immunofluorescent staining, as described previously [18]. Skin sections were incubated overnight at 4°C with anti‐PCNA antibody (sc‐56, 1:100), anti‐CK‐17 antibody (sc‐393002, 1:200) and anti‐VEGFR2 antibody (9698S, 1:200), then with FITC‐labelled goat anti‐rabbit IgG secondary antibody (1:1000) for 1 h at 37°C. Nuclei were stained with DAPI solution for an additional 10 min at room temperature. Imaging was performed using a fluorescence microscope and analysed with ImageJ. For PCNA analysis, fluorescence images were re‐evaluated with reference to DAPI nuclear counterstaining. PCNA‐positive nuclei were defined as nuclei showing spatial co‐localisation of PCNA fluorescence with DAPI staining. Non‐nuclear fluorescence and signals without clear overlap with DAPI‐positive nuclei were excluded from the PCNA‐positive nuclei count. The percentage of PCNA‐positive nuclei was calculated as the number of PCNA‐positive/DAPI‐positive nuclei divided by the total number of DAPI‐positive nuclei ×100. For fluorescence intensity measurements, hair follicle regions were excluded from the analysed areas. Measurements were obtained at the animal level (*n* = 7 mice/group). PCNA MFI was retained as a complementary measure of overall fluorescence intensity, whereas CK‐17 and VEGFR2 immunofluorescence was quantified using MFI.

### 2.8 Immunohistochemical (IHC) Staining

A previously established procedure was used to stain paraffin‐embedded sections with IHC [16]. Sections were incubated overnight with primary antibodies against NLRP3 (E‐AB‐65952, 1:150), ASC/TMS1 (E‐AB‐63530, 1:150), IL‐1β (sc‐52012, 1:200) and caspase‐1 (sc‐392736, 1:200). The following day, biotinylated secondary antibodies (TP‐125‐BN) and streptavidin peroxidase (TS‐125‐h) were applied, followed by DAB substrate for 3–5 min. After Mayer's haematoxylin counterstaining, slices were photographed with an Olympus BX53 microscope (DP74 camera, CellSens Entry, Olympus, Japan). ImageJ was used to quantify immunoreactivity for each antibody across 10 randomly selected fields from 3 to 5 slices per mice/group [16].

### 2.9 Real‐Time Quantitative Polymerase Chain Reaction (qPCR)

Total RNA was extracted using the TRIzol method, and cDNA was synthesised using the iScript cDNA Synthesis Kit (Bio‐Rad, Cytiva, GE27‐9261‐01). Quantitative real‐time PCR (qRT‐PCR) was performed using SYBR Green chemistry on a StepOnePlus Real‐Time PCR System (Applied Biosystems). *Actb* (β‐actin) was used as the reference gene for normalisation. Relative gene expression was calculated using the 2^−ΔΔ*Ct*^ method, with the control group used as the calibrator. Fold changes in target gene expression were expressed relative to the control group. The forward and reverse primer sequences and corresponding GenBank accession numbers for all genes analysed by qRT‐PCR are provided in Supporting Table S1.

### 2.10 Enzyme‐Linked Immunosorbent Assay (ELISA)

Serum levels of IL‐17A (BT Lab, E1143Mo) and IL‐1β (BT Lab, E0192Mo) were quantified using commercial ELISA kits following the manufacturers' protocols. Blood samples were collected post‐sacrifice and centrifuged to obtain serum for analysis.

### 2.11 Statistical Analysis

Sample size was estimated using G*Power to achieve 95% statistical power based on effect sizes reported in previous studies. For analyses involving multiple microscopic images, fields, or regions from the same animal, measurements were averaged to obtain a single animal‐level value before statistical analysis. The individual mouse, rather than each microscopic image, field, or region, was considered the independent experimental unit.

All statistical analyses were performed using GraphPad Prism version 8.0 (GraphPad Software, San Diego, CA, USA). Data distribution was assessed using the Shapiro–Wilk test. For normally distributed datasets, comparisons among multiple groups were performed using one‐way ANOVA followed by Tukey's multiple‐comparison test. For longitudinal measurements, two‐way repeated‐measures ANOVA followed by Bonferroni's multiple‐comparison test was used. For datasets that did not meet the assumption of normality, the Kruskal–Wallis test, followed by Dunn's multiple‐comparison test, was used for multiple‐group comparisons. For two‐group comparisons, an unpaired two‐tailed Student's *t*‐test was used for normally distributed data, whereas the Mann–Whitney *U* test was used for non‐normally distributed data. Data are presented as mean ± standard error of the mean (SEM), consistent with the graphical presentation of the experimental datasets. A *p*‐value < 0.05 was considered statistically significant, with significance levels defined as *p < 0.05, **p < 0.01, ***p < 0.001 and ****p < 0.0001.

## 3 Results

### 3.1 Body Weight Changes

IMQ‐induced inflammation is known to cause water loss and body weight reduction [22]. In this study, daily monitoring showed significant weight loss in the IMQ group compared with the control and IMQ + LOG groups (Figure S1, ****p < 0.0001). No significant weight change was observed in the other groups, and no animals died during the experiment. These findings demonstrate that LOG administration was associated with attenuation of IMQ‐induced body weight loss during the experimental period.

**Figure 1:**
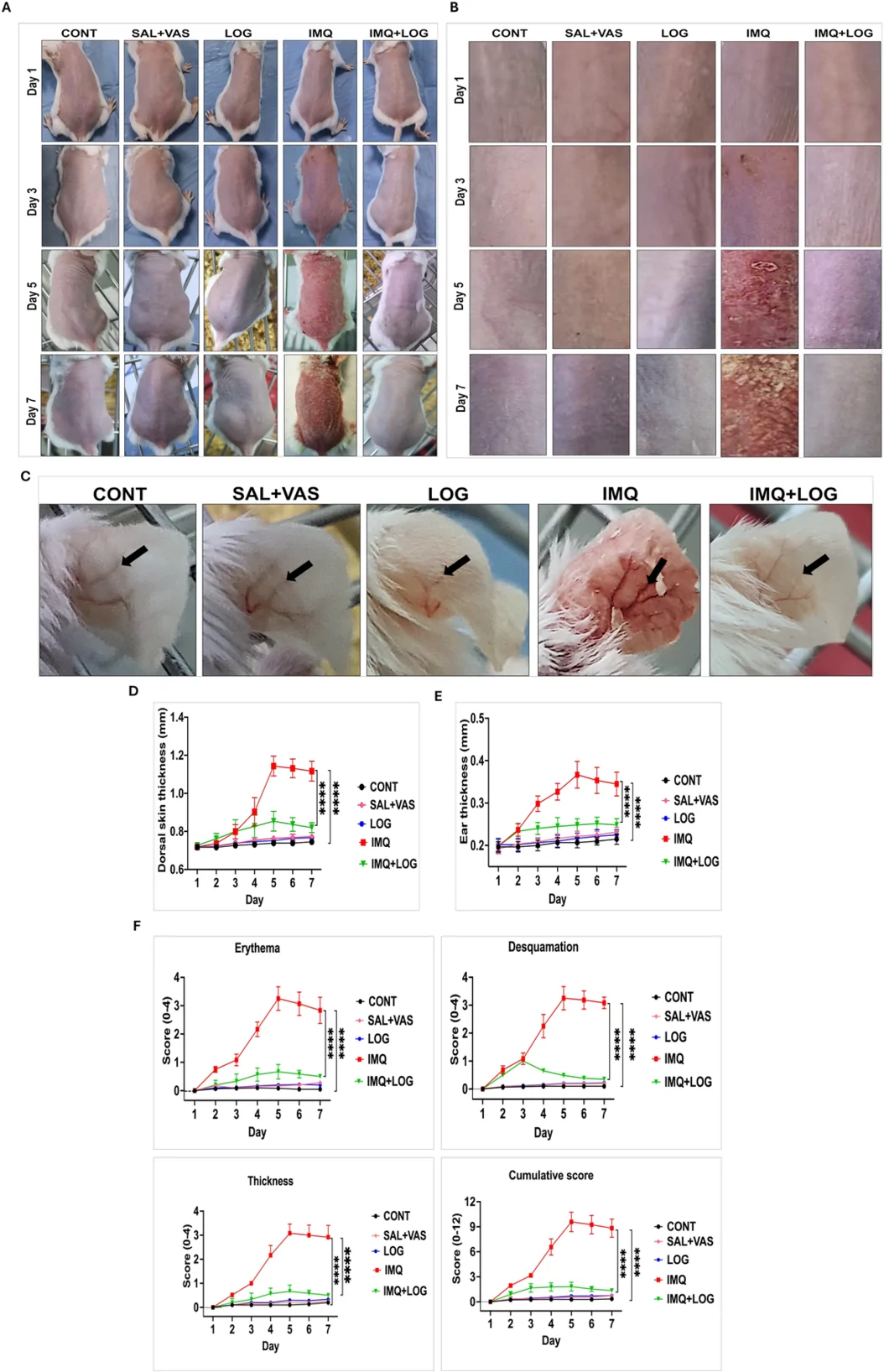
Representation of macroscopic changes and PASI scores in the dorsal and ear skin. (A) Photographs depicting the degree of severity of psoriatic inflammation on days 1, 3, 5 and 7 from experimental groups. (B) Zoomed images showing the severity of psoriatic inflammation on Days 1, 3, 5 and 7 of the dorsal skin. (C) Macroscopic presence of the right ear. Black arrows indicate superficial vessels. (D) Graph of dorsal skin thickness (mm) changes. (E) Graph of ear skin thickness (mm) alterations. (F) Effects of LOG on erythema, scaling, dorsal skin thickness and total cumulative PASI scores. Macroscopic parameters and PASI scores were analysed using two‐way repeated‐measures ANOVA followed by Bonferroni's multiple‐comparison test. Data are presented as mean ± SEM. Statistical significance was defined as ****p < 0.0001, compared with the IMQ group. (Cont: Control; IMQ: Imiquimod; LOG: Loganin; SAL + VAS: Saline + Vaseline).

### 3.2 Macroscopic Observations and Measurement of Skin Thickness

Topical IMQ application for 7 consecutive days induced psoriatic skin changes characterised by erythema, scaling, and increased skin thickness. Compared with control groups, IMQ produced a marked, progressive increase in dorsal and ear skin thickness, accompanied by erythema consistent with vascular dilatation (Figure 1A–E). Administration of LOG markedly attenuated IMQ‐induced macroscopic manifestations. Mice in the IMQ + LOG group showed significantly less skin thickening and erythema than those in the IMQ group (Figure 1A–E). Longitudinal measurements showed a time‐dependent increase in skin thickness after IMQ application (****p < 0.0001), whereas concurrent LOG significantly limited this progression over the experimental period (****p < 0.0001). No statistically significant differences were observed among the control, SAL + VAS, or LOG‐only groups (*p* > 0.05). Consistent with these findings, PASI scores, including erythema, scaling and thickness subscores (0–4), were significantly higher in the IMQ group than in controls (****p < 0.0001). LOG administration significantly reduced total PASI scores and individual subscores in the IMQ + LOG group (****p < 0.0001; Figure 1F). By contrast, PASI scores remained unchanged in control, SAL + VAS, and LOG‐only groups throughout the study period (*p* > 0.05). These results demonstrate that LOG administration significantly attenuated the clinical manifestations of IMQ‐induced psoriasis‐like skin inflammation, as reflected by reduced skin thickness and PASI scores.

### 3.3 Loganin Alleviates IMQ‐Induced Psoriatic Pathologic Features and Epidermal Thickness

Histopathological evaluation of H&E‐stained dorsal and ear skin sections showed preserved epidermal and dermal architecture in control groups. By contrast, IMQ administration induced prominent psoriatic skin changes, including marked acanthosis, parakeratosis, dermal inflammatory cell infiltration and vascular dilatation. The presence of Munro microabscesses and dense accumulations of immune cells, particularly in the perivascular and reticular dermis, further confirmed robust inflammatory activation in the IMQ group. These histopathological abnormalities were markedly attenuated in the IMQ + LOG group (Figure 2A–C). Consistent with these observations, epidermal thickness, an established histopathological hallmark of the psoriasis mouse model [4], was significantly increased after IMQ application compared with the control groups, whereas LOG administration produced a pronounced reduction in epidermal thickening (Figure 2D,E, ****p < 0.0001). No statistically significant differences were observed among the control groups (*p* > 0.05). Moreover, IMQ led to a significant increase in Baker scores, reflecting greater histopathological severity, whereas LOG significantly reduced these scores in the IMQ + LOG group (Kruskal–Wallis test followed by Dunn's multiple‐comparison test, ****p < 0.0001; Figure 2F). Collectively, these findings demonstrate that LOG administration significantly attenuated IMQ‐induced epidermal alterations and associated histopathological inflammatory changes. MT staining revealed no significant differences in dermal collagen fibre density in the dorsal and right ear skin of all groups (Figure S2A–C). These findings show that topical IMQ application, with or without LOG, does not alter collagen fibre density in the analysed skin regions.

**Figure 2:**
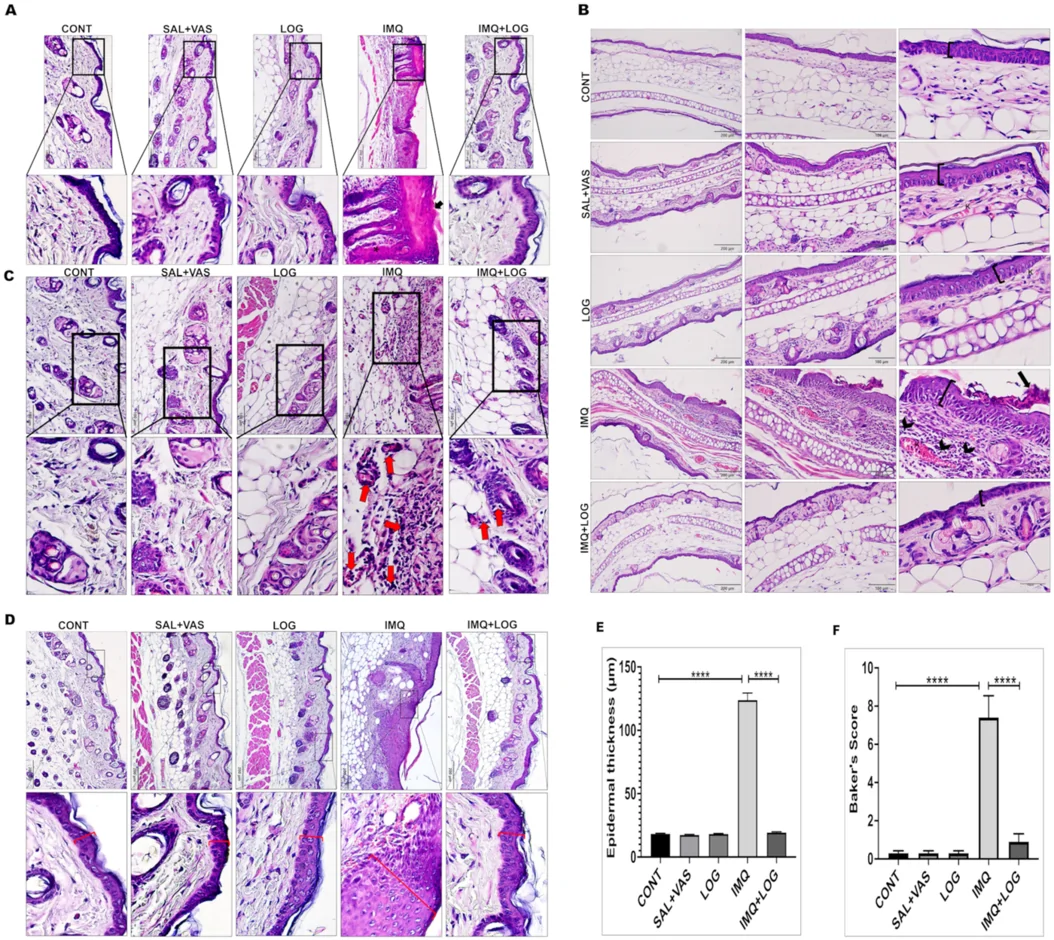
Effects of Loganin on histopathological alterations and histomorphometric parameters in dorsal and ear skin. (A) Microscopic image of hyperkeratosis, parakeratosis and acanthosis. Hyperkeratosis and parakeratosis are indicated by a black arrow and acanthosis by an asterisk (H&E staining, Scale bar: 100 μm; Magnification: 40×). (B) Microscopic images of the skin of the right ear. A bracket indicates epidermal thickness, Munro's microabscess by a thick arrow, lymphatic infiltration by an arrowhead, and capillary vessels. (H&E staining; Scale bar: 200 μm, 100 μm, 10 μm; Magnification: 10×, 20×, 40×). (C) Microscopic images of immune cell infiltration in the dorsal skin. Immune cell infiltration in intravascular and perivascular areas (red arrows) (H&E staining, Scale bar: 100 μm; Magnification: 40×). (D) H&E staining of microscopic images of epidermal thickness in dorsal skin sections. Epidermal thickness (bracket), (H&E staining, Scale bar: 200 μm; Magnification: 20×). (E) Graph of epidermal thickness averages of all groups. (F) Baker histopathological scores were analysed using the Kruskal–Wallis test followed by Dunn's multiple‐comparison test. Epidermal thickness measurements were analysed using one‐way ANOVA followed by Tukey's multiple‐comparison test after confirmation of normality. For epidermal thickness measurements, six non‐overlapping images from different tissue regions were evaluated per mouse, and the results were averaged to obtain a single animal‐level value. Data are presented as mean ± SEM (*n* = 7 mice/group). Statistical significance was defined as ****p < 0.0001 compared with the IMQ group. (Cont: Control; IMQ: Imiquimod; LOG: Loganin; SAL + VAS: Saline + Vaseline).

#### 3.3.1 LOG Attenuates IMQ‐Induced Vascular Alterations in Psoriatic Skin

IMQ markedly increased dermal vascularisation, vessel diameter and total vascular area compared with the control group (****p < 0.0001). Co‐administration of LOG significantly reduced these IMQ‐induced vascular changes, including vessel diameter and vascular area (****p < 0.0001). In parallel, microvascular area density was significantly increased in the IMQ group and reduced following LOG administration (Kruskal–Wallis test followed by Dunn's multiple‐comparison test, ****p < 0.0001; Figure 3E). These results demonstrate that LOG administration significantly attenuated IMQ‐associated dermal vascular alterations, as evidenced by reductions in vessel diameter, vascular area and microvascular area density.

**Figure 3:**
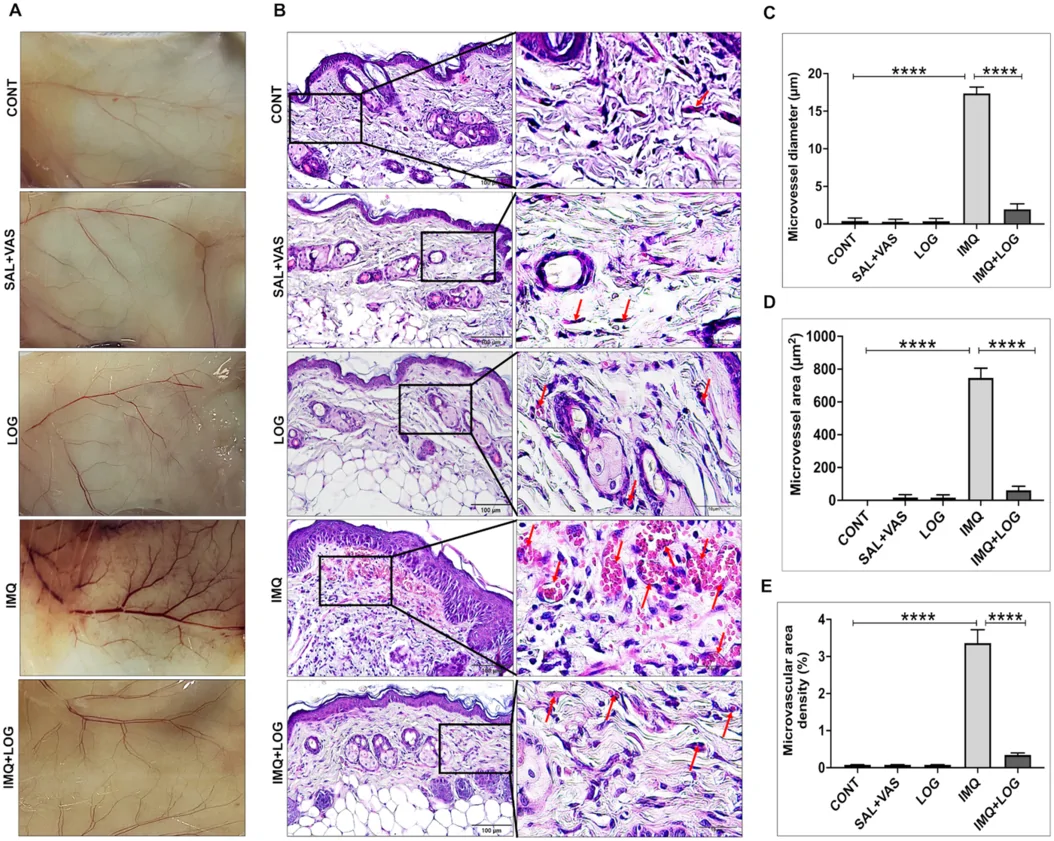
Macroscopic and microscopic representations of vascular areas in all groups. (A) Macroscopic image of subcutaneous vascular areas, (B) Microscopic image of vascular areas in H&E‐stained sections of the dorsal skin. Zoom in on images of the corresponding areas in the square. Vascular areas in the dermis (red arrow) (H&E staining, Scale bar: 100 μm, inset square 10 μm; Magnification: 40×). (C) Graph of microvessel diameter (μm) averages, (D) Graph of microvessel area (μm^2^) averages, (E) Graph of microvascular area density (%). For vascular morphometric measurements, 10 non‐overlapping dorsal skin regions were evaluated per mouse, and the results were averaged to obtain a single animal‐level value prior to statistical analysis. Vessel diameter and vascular area were analysed using one‐way ANOVA followed by Tukey's multiple‐comparison test, whereas microvascular area density was analysed using the Kruskal–Wallis test followed by Dunn's multiple‐comparison test. Data are presented as mean ± SEM (*n* = 7 mice/group). Statistical significance was indicated by ****p < 0.0001, versus the IMQ group. (Cont: Control; IMQ: Imiquimod; LOG: Loganin; SAL + VAS: Saline + Vaseline).

#### 3.3.2 LOG Reduces Epidermal and Dermal Volume Densities in IMQ‐Induced Psoriatic Skin

Stereological analysis revealed a significant increase in epidermal and dermal volume densities in the IMQ group compared with the control group (**p < 0.01, Figure S3A–E). LOG markedly reduced the IMQ‐induced increases (**p < 0.01). When expressed as relative volume density ratios calculated independently within the epidermal and dermal layers, IMQ exposure increased the epidermal/total skin and dermal/total skin ratios (**p < 0.01, Figure S3C,D), whereas LOG significantly attenuated these changes. These findings demonstrate that LOG administration significantly attenuated the IMQ‐induced increases in epidermal and dermal volume densities, consistent with reduced structural alterations in the dorsal skin.

### 3.4 LOG Attenuates PCNA‐Positive Nuclear Staining and CK‐17 and VEGFR2 Immunoreactivity in IMQ‐Induced Psoriatic Skin

IF analysis demonstrated marked alterations in PCNA, CK‐17 and VEGFR2 immunoreactivity following IMQ exposure (Figure 4A–E). Evaluation of PCNA immunofluorescence based on its co‐localization with DAPI positive nuclei revealed a significantly higher proportion of PCNA‐positive nuclei in the IMQ group compared with the control groups (****p < 0.0001; Figure 4A,B). Notably, PCNA‐positive nuclei appeared to be more prominent in the basal epidermal layer. LOG administration significantly reduced the proportion of PCNA‐positive nuclei in the IMQ + LOG group compared with the IMQ group (****p < 0.0001; Figure 4B). Consistent with the nuclear‐based analysis, overall PCNA mean fluorescence intensity (MFI) was markedly increased following IMQ exposure and significantly reduced following LOG administration (****p < 0.0001; Figure 4C).

**Figure 4:**
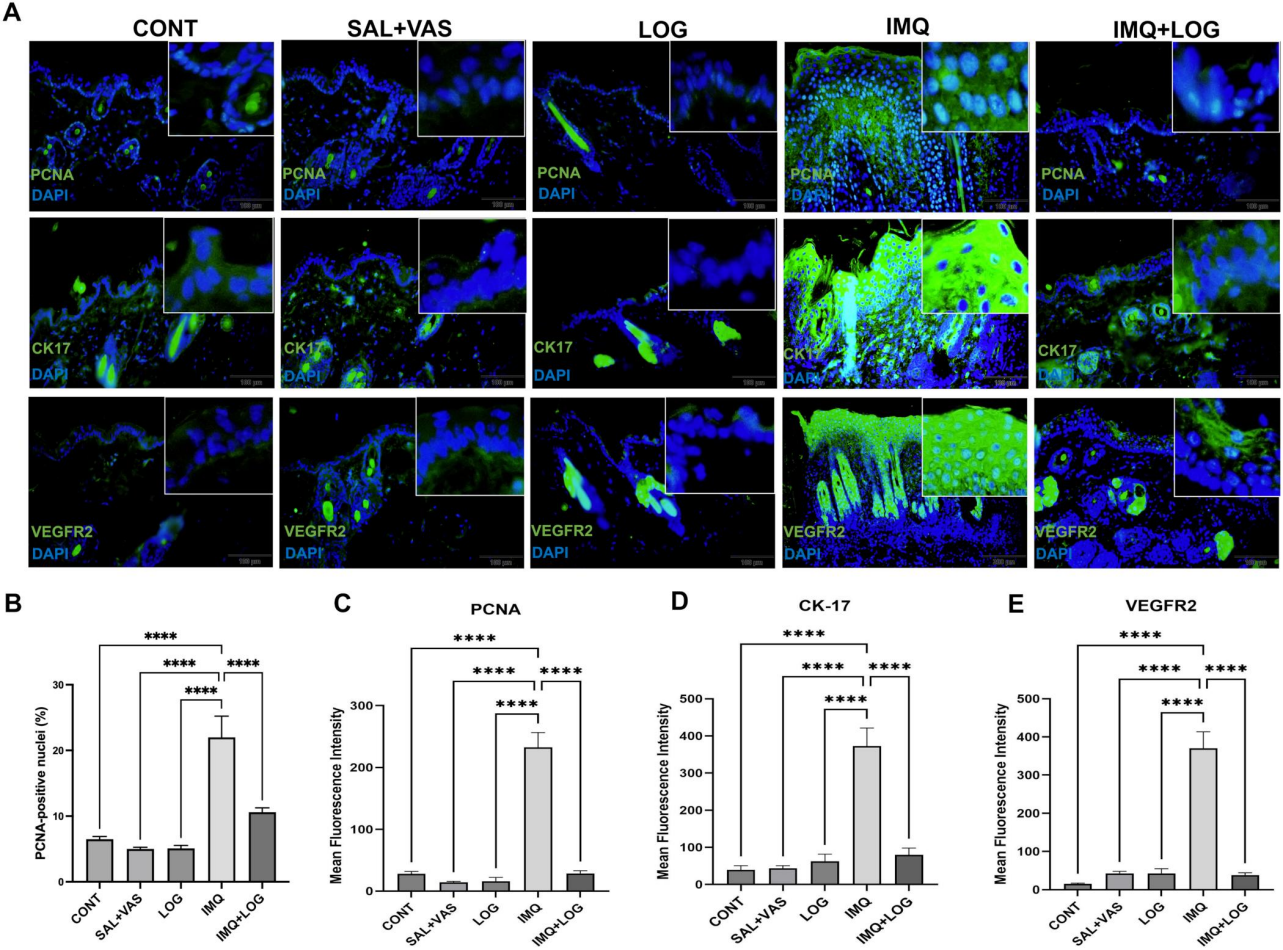
Immunofluorescence analysis of PCNA, CK‐17 and VEGFR2 in the dorsal skin. (A) Representative immunofluorescence images showing PCNA (green), CK‐17 (green) and VEGFR2 (green), with nuclei counterstained with DAPI (blue). Scale bar = 100 μm. (B) Percentage of PCNA‐positive nuclei, calculated as the number of PCNA‐positive/DAPI‐positive nuclei relative to the total number of DAPI‐positive nuclei ×100. Only PCNA fluorescence spatially co‐localising with DAPI‐positive nuclei was considered nuclear PCNA positivity; non‐nuclear fluorescence was excluded from this analysis. (C) Mean fluorescence intensity (MFI) of PCNA, retained as a complementary measure of overall PCNA immunofluorescence. (D) MFI of CK‐17 immunofluorescence. (E) MFI of VEGFR2 immunofluorescence. Data are presented as mean ± SEM (*n* = 7 mice/group). Statistical comparisons were performed using one‐way ANOVA followed by Tukey's multiple‐comparison test. Statistical significance was defined as ****p < 0.0001 for the comparisons indicated in the figure. (Cont: Control; IMQ: Imiquimod; LOG: Loganin; SAL + VAS: Saline + Vaseline).

CK‐17 immunoreactivity was also markedly increased in IMQ‐treated skin compared with the control groups (****p < 0.0001), whereas LOG administration significantly reduced CK‐17 MFI in the IMQ + LOG group compared with the IMQ group (****p < 0.0001; Figure 4D). Similarly, VEGFR2 immunoreactivity was significantly increased following IMQ exposure (****p < 0.0001) and was markedly reduced in the IMQ + LOG group (****p < 0.0001; Figure 4E). Overall, these findings demonstrate that LOG administration was associated with a significantly lower proportion of PCNA‐positive nuclei together with reduced overall PCNA, CK‐17 and VEGFR2 immunoreactivity in IMQ‐induced psoriatic skin.

### 3.5 LOG Reduces the Expression of NLRP3 Inflammasome‐Associated Components in IMQ‐Induced Skin Inflammation

Immunohistochemical analysis showed significantly increased immunoreactivity for NLRP3, ASC, caspase‐1 and IL‐1β in the IMQ‐treated group compared with the control group (Figure 5A,B, ****p < 0.0001). In contrast, LOG administration significantly reduced the immunoreactivity of all four markers in the IMQ + LOG group compared with the IMQ group (Figure 5A,B, ****p < 0.0001). These findings demonstrate that LOG administration is associated with reduced expression of NLRP3 inflammasome‐associated components in IMQ‐induced skin inflammation.

**Figure 5:**
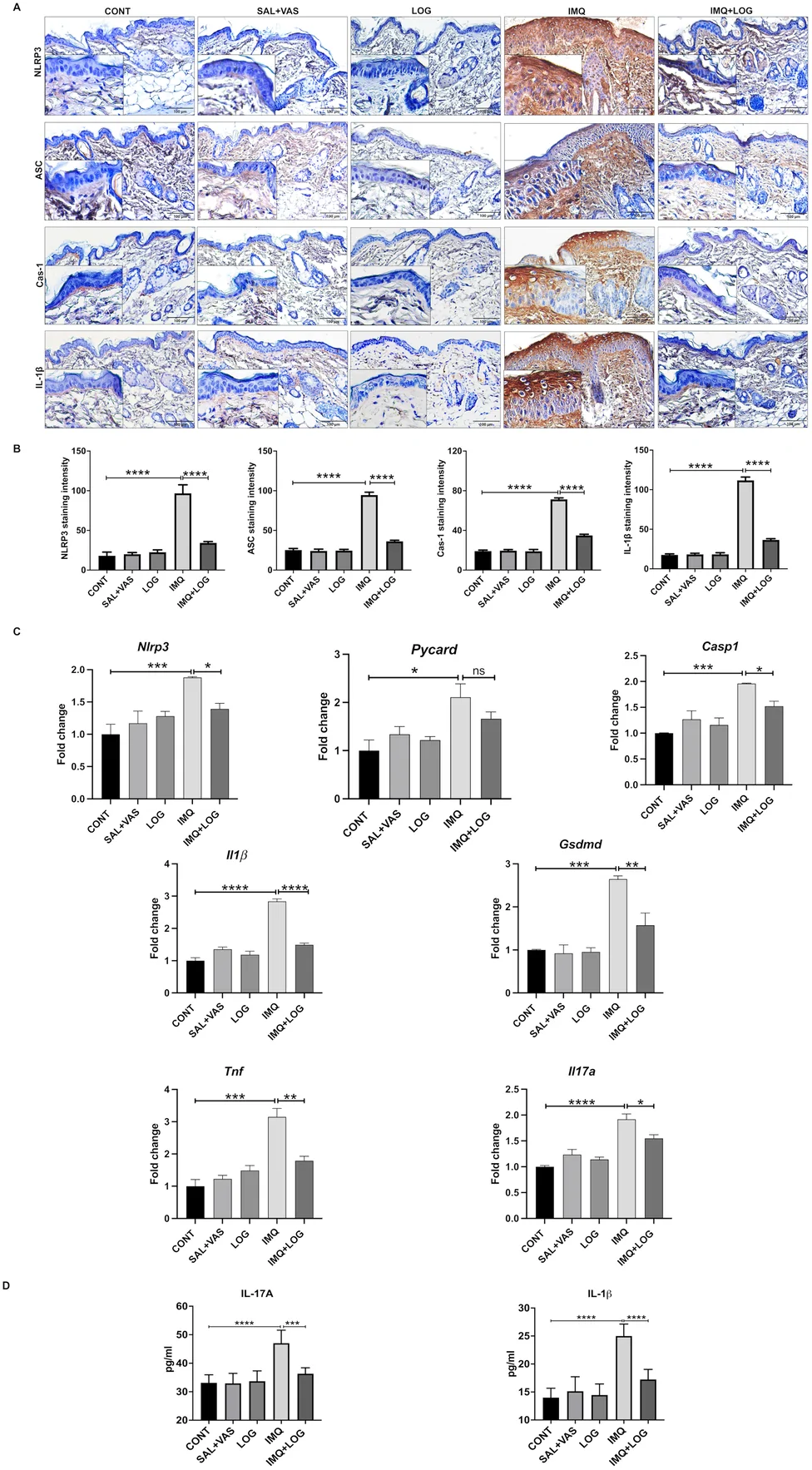
Effects of Loganin on NLRP3 inflammasome‐associated molecular markers in the IMQ‐induced psoriasis mouse model. (A) IHC staining of NLRP3, ASC, caspase‐1 and IL‐1β immunoreactivity in dorsal skin sections (brown‐stained areas) (Scale bar: 100 μm; Magnification: 40×), (B) Graphs of NLRP3, ASC, caspase‐1, and IL‐1β immunoreactivity. (C) Relative mRNA expression analysis of *Nlrp3, Pycard, Casp1, Il1b, Gsdmd, Tnf* and *Il17a*. (D) Enzyme‐linked immunosorbent assay (ELISA) results of IL‐1β and IL‐17A proteins of the groups (pg/mL). IHC immunoreactivity, relative mRNA expression and serum cytokine data were analysed using one‐way ANOVA followed by Tukey's multiple‐comparison test after confirmation of normality. Data are presented as mean ± SEM (*n* = 7 mice/group). Statistical significance was defined as *p < 0.05, **p < 0.01, ***p < 0.001 and ****p < 0.0001 versus the IMQ group. (Cas‐1: Caspase‐1; Cont: Control; IMQ: Imiquimod; LOG: Loganin; SAL + VAS: Saline + Vaseline).

### 3.6 Determination of *Nlrp3* Inflammasome and Psoriasis‐Associated Gene Expression Levels by qRT‐PCR

qRT‐PCR analysis revealed significantly increased mRNA expression of *Nlrp3, Pycard, Casp1, Il1b, Gsdmd, Tnf* and *Il17a* in the IMQ group compared with the control group (* *p* < 0.05, **p < 0.01, ***p < 0.001 and ****p < 0.0001; Figure 5C). These IMQ‐induced increases were significantly attenuated in the IMQ + LOG group. No significant differences were observed among the control, SAL + VAS, and LOG‐only groups. Collectively, these results demonstrate that LOG administration was associated with reduced transcriptional expression of multiple inflammation‐ and inflammasome‐associated genes that were upregulated following IMQ exposure.

### 3.7 Effects of LOG on Psoriasis‐Associated Pro‐Inflammatory Cytokine Levels

The IMQ application significantly increased serum levels of the pro‐inflammatory cytokines IL‐17A and IL‐1β compared with the control groups (Figure 5D, ****p < 0.0001). LOG significantly decreased the levels of IL‐17A and IL‐1β in the IMQ + LOG group compared with the IMQ group (Figure 5D, ***p < 0.001). These findings demonstrate significantly lower circulating levels of IL‐17A and IL‐1β following LOG administration in IMQ‐treated mice.

## 4 Discussion

Psoriasis is a chronic immune‐mediated inflammatory skin disorder characterised by keratinocyte hyperproliferation and immune cell infiltration. Dysregulated interactions between keratinocytes and immune cells, together with aberrant inflammatory signalling, play a central role in disease pathogenesis [18]. Increasing evidence indicates that inflammasome‐associated pathways, including NLRP3, are altered in psoriatic skin; however, these alterations are now considered part of a broader inflammatory network rather than isolated primary drivers of disease [23]. In this context, plant‐derived bioactive compounds with anti‐inflammatory properties have attracted increasing attention for their multitarget potential and favourable safety profiles [4]. In the present study, LOG was evaluated in an IMQ‐induced psoriasis mouse model to investigate its effects on inflammatory signalling and the associated tissue responses.

IMQ, a TLR7/8 agonist, induces robust innate immune activation, resulting in epidermal hyperplasia, erythema and scaling, features that closely resemble those of human psoriasis [5]. Consistent with previous studies, IMQ administration produced marked clinical and histopathological changes, accompanied by body weight loss [24, 25, 26]. LOG administration was associated with lower PASI‐like scores and attenuation of IMQ‐associated body‐weight loss, consistent with reduced clinical disease severity during the experimental period. Epidermal hyperplasia is a hallmark of psoriasis and reflects dysregulated keratinocyte proliferation and differentiation within an inflammatory microenvironment [18]. PCNA is widely used as a nuclear marker of proliferative activity, whereas CK17 is markedly upregulated in activated psoriatic keratinocytes and is associated with epidermal hyperproliferation and disease‐associated keratinocyte remodelling [27]. Consistent with previous reports, IMQ‐induced increases in PCNA expression and epidermal thickness are associated with keratinocyte hyperproliferation in psoriatic skin [28]. In the present study, nuclear PCNA analysis, based on co‐localisation with DAPI, showed an increased proportion of PCNA‐positive nuclei after IMQ exposure, accompanied by increased epidermal thickness and histopathological severity. LOG administration was associated with a reduction in the proportion of PCNA‐positive nuclei, together with attenuation of epidermal thickening and CK17 immunoreactivity. These parallel changes are consistent with attenuation of the abnormal epidermal proliferative response induced by IMQ. Importantly, the present findings do not establish a direct antiproliferative effect of LOG on keratinocytes. Because keratinocyte proliferation was assessed within the inflammatory microenvironment of IMQ‐treated skin, the reduction in PCNA‐positive nuclei and epidermal thickening may, at least in part, be secondary to the attenuation of inflammatory signalling following LOG administration. This interpretation is supported by the concomitant reductions in inflammatory cytokines and keratinocyte‐associated alterations observed in the IMQ + LOG group. Nevertheless, a direct effect of LOG on keratinocyte proliferative activity cannot be excluded based on the present in vivo findings. Studies using isolated primary keratinocytes or keratinocyte cell lines, together with direct assessment of cell‐cycle and proliferation‐related endpoints, will be required to distinguish direct antiproliferative effects from those occurring secondary to modulation of the inflammatory microenvironment.

IL‐17A is an important component of the inflammatory environment that promotes keratinocyte activation and has been linked to increased CK17 expression in psoriatic skin [29, 30]. Therefore, the concomitant reduction in circulating IL‐17A levels and CK17 immunoreactivity observed in the IMQ + LOG group is biologically consistent with attenuation of IL‐17‐associated keratinocyte activation. However, because the present study did not directly assess IL‐23 signalling, Th17‐cell activity, or the causal relationship between IL‐17A and CK17 expression, these findings should not be interpreted as direct evidence that LOG inhibits the IL‐23/Th17/keratinocyte axis. The reductions in circulating IL‐17A and IL‐1β following LOG administration should also be considered in the context of psoriasis's multicellular inflammatory network. IL‐17A is predominantly associated with Th17 responses but can also be produced by other immune cell populations, particularly γδ T cells, which contribute substantially to IL‐17‐driven inflammation in the IMQ model. IL‐1β may originate from activated myeloid cells, including macrophages and dendritic cells, as well as from inflammatory responses involving keratinocytes. These cytokines can participate in reciprocal immune–keratinocyte signalling that promotes epidermal activation and sustains psoriatic inflammation [31, 32]. Accordingly, the lower circulating levels of IL‐17A and IL‐1β observed in the IMQ + LOG group are consistent with an attenuated inflammatory state. However, because cytokines were measured in serum and cell‐specific analyses were not performed, the present study cannot determine which cellular populations account for these changes.

Previous studies in inflammatory models have reported that LOG can modulate NF‐κB‐associated signalling and related inflammatory pathways that regulate pro‐inflammatory cytokine production [10, 15]. NF‐κB, MAPK and STAT3 signalling are also well‐established contributors to inflammatory amplification and keratinocyte activation in psoriasis [33, 34]. Recent experimental evidence further supports the relevance of these pathways in psoriasis. Li et al. demonstrated that Pulsatilla saponin attenuated keratinocyte proliferation and IMQ‐induced psoriasis‐like inflammation in association with modulation of NF‐κB and JAK1/STAT3 signalling [35]. Similarly, Walvekar et al. reported that Biochanin A ameliorated IMQ‐induced psoriasis‐like skin inflammation and reduced activation of NF‐κB and MAPK signalling, including ERK1/2, p38, JNK and c‐Jun [36]. Previous pharmacological studies using pathway‐directed inhibitors in IMQ‐induced psoriasis models have further demonstrated that suppression of NF‐κB‐ and STAT3‐associated signalling can attenuate inflammatory and epidermal responses [37, 38]. These findings provide a relevant mechanistic context for the reductions in inflammatory and keratinocyte‐associated markers observed following LOG administration in the present study. However, because NF‐κB, MAPK and STAT3 activation were not directly assessed, our findings cannot establish these pathways as mediators of LOG activity. They should therefore be considered plausible mechanistic contexts rather than experimentally demonstrated mechanisms. Future studies incorporating pathway‐specific inhibitors together with assessment of NF‐κB activation, MAPK phosphorylation and STAT3 phosphorylation will be required to determine whether these signalling pathways contribute directly to the effects of LOG in IMQ‐induced skin inflammation.

Psoriasis is characterised by pronounced microvascular alterations, including aberrant angiogenesis and endothelial activation [39, 40]. VEGFR2 regulates endothelial proliferation and inflammatory angiogenesis [39]. In this context, the increased VEGFR2 immunoreactivity observed in IMQ‐treated skin is consistent with the aberrant vascular responses described in psoriatic inflammation. Accordingly, the reduction in VEGFR2 immunoreactivity following LOG administration was accompanied by attenuation of inflammation‐associated vascular alterations. Together, the reductions in VEGFR2 immunoreactivity and vascular morphometric parameters indicate attenuation of IMQ‐associated vascular alterations following LOG administration. These vascular changes were accompanied by reduced epidermal thickening and attenuation of dermal structural alterations, as further supported by the stereological findings. An important strength of the present study is the quantitative stereological assessment of epidermal and dermal structural changes. IMQ‐induced increases in epidermal and dermal volume densities were accompanied by increased epidermal thickness, histopathological severity, inflammatory infiltration, and alterations in keratinocyte‐ and vascular‐associated markers. Conversely, the reduction in these stereological parameters following LOG administration paralleled the attenuation of epidermal thickening, PCNA and CK‐17 immunoreactivity, inflammatory changes and vascular alterations. The convergence of stereological, histopathological and molecular findings therefore provides complementary quantitative evidence that LOG administration is associated with attenuation of IMQ‐induced tissue remodelling. Importantly, the absence of significant changes in collagen fibre density suggests that these structural alterations predominantly involve epidermal expansion, inflammatory dermal changes and vascular responses rather than substantial extracellular matrix remodelling during the experimental period. Consistent with previous studies showing that psoriasis typically does not involve marked collagen degradation [41, 42, 43], collagen fibre density remained unchanged between groups, suggesting that the effects of LOG are linked to inflammation and vascular modulation rather than direct extracellular matrix alteration.

Recent evidence further supports the involvement of NLRP3 inflammasome‐associated signalling in psoriasis and indicates that inflammasome activity operates within a broader inflammatory network involving keratinocytes, myeloid cells, neutrophils and cytokine‐mediated inflammatory circuits [44, 45]. Canonical NLRP3 inflammasome activation is generally described as a sequential process involving priming and activation. During the priming phase, inflammatory signals, frequently through NF‐κB‐associated signalling, increase the transcriptional availability of NLRP3 and pro‐IL‐1β [2]. Following an appropriate activation signal, NLRP3 undergoes oligomerisation and recruits the adaptor protein ASC, which facilitates the recruitment and proximity‐induced activation of pro‐caspase‐1. Activated caspase‐1 subsequently processes pro‐IL‐1β into its mature form and can cleave gasdermin D (GSDMD), thereby linking inflammasome activation to cytokine release and pyroptotic signalling. In psoriasis, IL‐1β generated within this inflammatory environment may further amplify immune–keratinocyte crosstalk and promote IL‐17‐associated inflammatory responses, thereby contributing to the persistence of psoriatic inflammation [44]. In the present study, IMQ exposure was associated with coordinated increases in NLRP3, ASC, caspase‐1 and IL‐1β immunoreactivity, together with increased transcriptional expression of Nlrp3, Pycard, Casp1, Il1b and Gsdmd. LOG administration was associated with reductions in these inflammasome‐related molecular readouts, accompanied by lower circulating IL‐1β and IL‐17A levels and attenuation of epidermal and inflammatory alterations. When considered within the canonical NLRP3 pathway, the coordinated changes across NLRP3, ASC, caspase‐1 and IL‐1β are consistent with attenuation of molecular responses associated with inflammasome signalling following LOG administration. The concomitant reduction in IL‐1β may also contribute to the broader attenuation of inflammatory amplification observed in IMQ‐treated skin. However, the present findings do not establish a sequential causal relationship among these molecular changes or demonstrate that LOG directly suppresses canonical NLRP3 inflammasome activation. Importantly, expression‐level measurements of NLRP3, ASC, caspase‐1 and IL‐1β alone cannot establish functional inflammasome assembly or activation. Although Gsdmd expression was assessed transcriptionally, ASC oligomerisation or speck formation, caspase‐1 cleavage/activity, mature IL‐1β and cleaved GSDMD were not directly evaluated. Furthermore, because NF‐κB activation was not assessed, the present study cannot determine whether the reductions in inflammasome‐associated markers following LOG administration result from modulation of upstream priming, inflammasome assembly/activation, or broader suppression of the inflammatory microenvironment. Accordingly, these findings should be interpreted as evidence that LOG attenuates molecular responses associated with the NLRP3 inflammasome rather than as direct demonstration of inflammasome inhibition. Future studies incorporating ASC oligomerisation, cleaved caspase‐1, mature IL‐1β, cleaved GSDMD, and pathway‐specific functional analyses will be required to determine whether LOG directly interferes with canonical NLRP3 inflammasome activation and its downstream pyroptotic signalling.

Several limitations of the present study should be considered when interpreting these findings. First, only female BALB/c mice were included; therefore, the potential influence of biological sex on the inflammatory response to IMQ and the effects of LOG could not be evaluated, and the findings should not be generalised to male animals without further investigation. Second, only a single site‐specific dosing regimen of LOG was examined. Although the selected doses were based on previous in vivo studies, the present design does not permit determination of a dose–response relationship, optimal effective dose, or therapeutic window. Third, LOG was administered subcutaneously to the dorsal and right ear regions rather than topically. Because the injections were performed at anatomical sites corresponding to the IMQ‐treated skin regions, the present design does not allow the relative contribution of local versus systemic LOG exposure to be distinguished. Moreover, topical administration may be more directly relevant to the treatment of a cutaneous disease. Future studies comparing subcutaneous and topical administration, together with assessment of cutaneous penetration and local bioavailability, will be required to better define the translational relevance of LOG. Finally, functional indices of NLRP3 inflammasome activation and upstream signalling pathways were not directly assessed. Future studies incorporating both sexes, dose‐ranging designs, alternative routes of administration and functional molecular analyses will be necessary to further define the efficacy, mechanism and translational potential of LOG in psoriasis. Taken together, these findings indicate that LOG may influence multiple inflammatory processes in the IMQ‐induced psoriasis mouse model. Further studies incorporating protein‐level assessments of upstream signalling pathways will be necessary to clarify the molecular basis of these outcomes.

## 5 Conclusion

The present study demonstrates that LOG attenuates IMQ‐induced psoriasis‐like skin inflammation in mice, as evidenced by reduced clinical severity, epidermal hyperplasia, inflammatory cell infiltration, keratinocyte‐associated alterations and microvascular changes.

These effects were accompanied by decreased IL‐17A and IL‐1β levels and reduced mRNA and immunohistochemical expression of NLRP3 inflammasome‐associated components, including NLRP3, ASC, caspase‐1 and IL‐1β. Collectively, these findings support an anti‐inflammatory effect of LOG and indicate that its administration attenuates multiple inflammatory and tissue responses in the IMQ‐induced psoriasis model. However, because functional indices of inflammasome activation, including inflammasome assembly, caspase‐1 activation, mature IL‐1β processing and GSDMD cleavage, were not directly assessed, the present findings should not be interpreted as evidence of direct NLRP3 inflammasome inhibition. Further studies incorporating functional inflammasome assays and upstream signalling analyses are warranted to define the molecular mechanisms underlying the anti‐inflammatory effects of LOG.

## Author Contributions

**Eda Acikgoz:** conceptualisation, data curation, formal analysis, investigation, methodology, project administration, supervision, validation, visualisation, writing – original draft, writing – review and editing. **Cafer Tas:** conceptualisation, data curation, formal analysis, investigation, methodology, visualisation. **Mustafa Cakir:** data curation, formal analysis, methodology, validation, visualisation, writing – original draft, writing – review and editing. **Seda Keskin:** data curation, formal analysis, methodology, visualisation, writing – original draft, writing – review and editing. All the authors read and approved the final manuscript. The authors declare that all data were generated in‐house, that no paper mill was used, and that no AI tool was used to generate text or figures.

## Ethics Statement

All animal studies were approved by the Animal Research Ethics Committee (2021/10‐14) of Van Yuzuncu Yil University, in accordance with the National Institutes of Health Guide for the Care and Use of Laboratory Animals and the ARRIVE guidelines.

## Conflicts of Interest

The authors declare no conflicts of interest.

## Supporting Information

Supplementary data associated with this article can be found in the online version.

## Supporting information


Supporting File

## Data Availability

The data will be available upon request to the corresponding author.

## References

[1] M. Wu , C. Dai , and F. Zeng , “Cellular Mechanisms of Psoriasis Pathogenesis: A Systemic Review,” Clinical, Cosmetic and Investigational Dermatology 16 (2023): 2503–2515, 10.2147/CCID.S420850. PMC10506593 37727872

[2] A. Shi , Y. Shu , K. Hu , S. Sudesh , and Y. Tu , “NLRP3‐inflammasome Related Genes as Emerging Biomarkers and Therapeutic Targets in Psoriasis,” Inflammation 48 (2025): 3366–3378, 10.1007/s10753-025-02271-y. PMC12596316 40029502

[3] A. Shahi , S. Afzali , A. Amirzargar , P. Mohaghegh , S. Salehi , and Y. Mansoori , “Potential Roles of Inflammasomes in the Pathophysiology of Psoriasis: A Comprehensive Review,” Molecular Immunology 161 (2023): 44–60, 10.1016/j.molimm.2023.06.007. 37481828

[4] M.‐C. Shih , C.‐L. Li , E.‐C. Liao , et al., “Inhibition of NLRP3 Inflammasome Activation by 3H‐1, 2‐Dithiole‐3‐Thione: A Potential Therapeutic Approach for Psoriasis Treatment,” International Journal of Molecular Sciences 24, no. 17 (2023): 13528, 10.3390/ijms241713528. PMC10487783 37686332

[5] L. Van der Fits , S. Mourits , J. S. A. Voerman , et al., “Imiquimod‐Induced Psoriasis‐Like Skin Inflammation in Mice Is Mediated via the IL‐23/IL‐17 Axis,” Journal of Immunology 182, no. 9 (2009): 5836–5845, 10.4049/jimmunol.0802999. 19380832

[6] Y. D. Xu , C. Cui , M. F. Sun , et al., “Neuroprotective Effects of Loganin on MPTP‐Induced Parkinson's Disease Mice: Neurochemistry, Glial Reaction and Autophagy Studies,” Journal of Cellular Biochemistry 118, no. 10 (2017): 3495–3510, 10.1002/jcb.26010. 28338241

[7] J. H. Jang , G. Yang , J. K. Seok , et al., “Loganin Prevents Hepatic Steatosis by Blocking NLRP3 Inflammasome Activation,” Biomolecules & Therapeutics 31, no. 1 (2022): 40–47, 10.4062/biomolther.2022.077. PMC9810450 36111592

[8] X. Kong , Y. Zhao , X. Wang , et al., “Loganin Reduces Diabetic Kidney Injury by Inhibiting the Activation of NLRP3 Inflammasome‐Mediated Pyroptosis,” Chemico‐Biological Interactions 382 (2023): 110640, 10.1016/j.cbi.2023.110640. 37473909

[9] W. Jiang , K. Ding , T. Gong , Z. Hu , R. Yue , and M. Yang , “Loganin and Morroniside: Therapeutic Effects and Molecular Mechanisms in Diabetes Mellitus and Its Complications—A Review,” European Journal of Pharmacology 1003 (2025): 177993, 10.1016/j.ejphar.2025.177993. 40714074

[10] X. Huang , X. Chen , Q. Li , B. Ni , and W. Wang , “Loganin Reduces Neuroinflammation and Promotes Functional Recovery After Spinal Cord Injury Through Inhibiting NF‐κB/NLRP3 Signalling,” Toxicon 232 (2023): 107202, 10.1016/j.toxicon.2023.107202. 37348819

[11] G. S. Girard , S. K. Moran , and S. R. Feldman , “Advertising Techniques and Biologic Treatment in Psoriasis,” in Business Ethics in the Healthcare Industry. Springer, 2026, 369–379.

[12] N. Ju , M. Shimamura , H. Hayashi , et al., “Preventative Effects of the Partial RANKL Peptide MHP1‐AcN in a Mouse Model of Imiquimod‐Induced Psoriasis,” Scientific Reports 9, no. 1 (2019): 15434, 10.1038/s41598-019-51681-0. PMC6817907 31659208

[13] C.‐H. Pan , C.‐Y. Xia , Y. Yan , et al., “Loganin Ameliorates Depression‐Like Behaviors of Mice via Modulation of Serotoninergic System,” Psychopharmacology 238, no. 11 (2021): 3063–3070, 10.1007/s00213-021-05922-8. 34342673

[14] Y. Li , Z. Li , L. Shi , et al., “Loganin Inhibits the Inflammatory Response in Mouse 3T3L1 Adipocytes and Mouse Model,” International Immunopharmacology 36 (2016): 173–179, 10.1016/j.intimp.2016.04.026. 27155393

[15] S. Liu , H. Shen , J. Li , et al., “Loganin Inhibits Macrophage M1 Polarization and Modulates sirt1/NF‐κB Signaling Pathway to Attenuate Ulcerative Colitis,” Bioengineered 11, no. 1 (2020): 628–639, 10.1080/21655979.2020.1774992. PMC8291865 33034242

[16] B. Baba , T. Ceylani , H. T. Teker , et al., “Therapeutic Potential of Young Plasma in Reversing Age‐Related Liver Inflammation via Modulation of NLRP3 Inflammasome and Necroptosis,” Biogerontology 26, no. 3 (2025): 117, 10.1007/s10522-025-10260-9. PMC12106525 40418410

[17] B. S. Baker , L. Brent , H. Valdimarsson , et al., “Is Epidermal Cell Proliferation in Psoriatic Skin Grafts on Nude Mice Driven by T‐Cell Derived Cytokines?,” British Journal of Dermatology 126, no. 2 (1992): 105–110, 10.1111/j.1365-2133.1992.tb07805.x. 1536776

[18] M. Cakir , S. Keskin , Y. Uyanikgil , S. Cakir , and E. Acikgoz , “Imiquimod‐Induced Psoriasis‐Like Inflammation Model in Calcium‐Differentiated Human Keratinocytes Mimics Psoriasis‐Associated Biomolecules and Signaling Pathways,” Archives of Dermatological Research 317, no. 1 (2025): 856, 10.1007/s00403-025-04336-2.

[19] V. Howard and M. Reed , Unbiased Stereology: Three‐dimensional Measurement in Microscopy. Garland Science, 2004.

[20] H. J. G. Gundersen , E. B. V. Jensen , K. Kiêu , and J. Nielsen , “The Efficiency of Systematic Sampling in Stereology—Reconsidered,” Journal of Microscopy 193, no. 3 (1999): 199–211, 10.1046/j.1365-2818.1999.00457.x. 10348656

[21] H. J. G. Gundersen and E. B. Jensen , “The Efficiency of Systematic Sampling in Stereology and Its Prediction,” Journal of Microscopy 147, no. 3 (1987): 229–263, 10.1111/j.1365-2818.1987.tb02837.x. 3430576

[22] Q. Li , W. Liu , S. Gao , Y. Mao , and Y. Xin , “Application of Imiquimod‐Induced Murine Psoriasis Model in Evaluating Interleukin‐17A Antagonist,” BMC Immunology 22, no. 1 (2021): 11, 10.1186/s12865-021-00401-3. PMC7844923 33509093

[23] M. Ciążyńska , I. Olejniczak‐Staruch , D. Sobolewska‐Sztychny , J. Narbutt , M. Skibińska , and A. Lesiak , “The Role of NLRP1, NLRP3, and AIM2 Inflammasomes in Psoriasis,” International Journal of Molecular Sciences 22, no. 11 (2021): 5898, 10.3390/ijms22115898. PMC8198493 34072753

[24] A. D. Griffith , A. K. Zaidi , A. Pietro , et al., “A Requirement for slc15a4 in Imiquimod‐Induced Systemic Inflammation and Psoriasiform Inflammation in Mice,” Scientific Reports 8, no. 1 (2018): 14451, 10.1038/s41598-018-32668-9. PMC6160456 30262916

[25] P. Zanvit , J. E. Konkel , X. Jiao , et al., “Antibiotics in Neonatal Life Increase Murine Susceptibility to Experimental Psoriasis,” Nature Communications 6, no. 1 (2015): 8424, 10.1038/ncomms9424. PMC4598725 26416167

[26] F. Salwa , M. Badanthadka , and L. D'Souza , “Differential Psoriatic Effect of Imiquimod on BALB/c and Swiss Mice,” Journal of Health and Allied Sciences NU 11, no. 03 (2021): 170–177, 10.1055/s-0041-1726681.

[27] X. Qiu , D. Hong , Z. Shi , et al., “TNF‐α Promotes CXCL‐1/8 Production in Keratinocytes by Downregulating Galectin‐3 Through NF‐κB and hsa‐miR‐27a‐3p Pathway to Contribute Psoriasis Development,” Immunopharmacology and Immunotoxicology 45, no. 6 (2023): 692–700, 10.1080/08923973.2023.2229510. 37358143

[28] C. Park , H. Lee , S. Jin , et al., “The Preventive Effect of Loganin on Oxidative Stress‐Induced Cellular Damage in Human Keratinocyte HaCaT Cells,” BioScience Trends 16, no. 4 (2022): 291–300, 10.5582/bst.2022.01116. 35691912

[29] L. Jin and G. Wang , “Keratin 17: A Critical Player in the Pathogenesis of Psoriasis,” Medicinal Research Reviews 34, no. 2 (2014): 438–454, 10.1002/med.21291. 23722817

[30] Y. Lin , W. Zhang , B. Li , and G. Wang , “Keratin 17 in Psoriasis: Current Understanding and Future Perspectives,” Seminars in Cell & Developmental Biology 128 (2022): 112–119, 10.1016/j.semcdb.2021.06.018. 34229948

[31] M. A. X. Tollenaere , J. Hebsgaard , D. A. Ewald , et al., “Signalling of Multiple Interleukin (IL)‐17 Family Cytokines via IL‐17 Receptor A Drives Psoriasis‐Related Inflammatory Pathways,” British Journal of Dermatology 185, no. 3 (2021): 585–594, 10.1111/bjd.20090. PMC8453543 33792895

[32] Y. Wang , F. Wei , Y. Guo , et al., “Smilacis Chinae Rhizoma Ethyl Acetate Fraction Alleviates IMQ‐Induced Psoriasis by Inhibiting IL‐17‐driven T‐Cell Immune Responses,” Journal of Ethnopharmacology 369 (2026): 121926, 10.1016/j.jep.2026.121926. 42219068

[33] A. Liu , B. Zhang , W. Zhao , Y. Tu , Q. Wang , and J. Li , “Catalpol Ameliorates Psoriasis‐Like Phenotypes via SIRT1 Mediated Suppression of NF‐κB and MAPKs Signaling Pathways,” Bioengineered 12, no. 1 (2021): 183–195, 10.1080/21655979.2020.1863015. PMC8806253 33323018

[34] L. Li , L. Chen , C. Zhang , W. Yao , Z. Li , and F. Tian , “Role of the STAT3 Signaling Pathway in Cell Proliferation and Inflammation in Psoriasis and Approaches for Targeted Therapies: A Review,” Medical Science Monitor 32 (2026): e952449, 10.12659/MSM.952449. PMC13114201 42015469

[35] J. Li , H. Qiu , S. Li , et al., “Pulsatilla Saponin Inhibits the Proliferation of Keratinocytes and Ameliorates Imiquimod‐Induced Psoriasis Through the NF‐κB and STAT3 Signaling Pathways,” Journal of Traditional and Complementary Medicine 15, no. 4 (2025): 356–368, 10.1016/j.jtcme.2024.04.001. PMC12266170 40677538

[36] K. P. Walvekar , S. K. Tirunavalli , A. C. Eedara , et al., “Biochanin A Ameliorates Imiquimod‐Induced Psoriasis‐Like Skin Inflammation in Mice by Modulating the NF‐κB and MAPK Signaling Pathways,” Inflammation 48, no. 3 (2025): 1125–1142, 10.1007/s10753-024-02103-5. 39017810

[37] N. Irrera , M. Vaccaro , A. Bitto , et al., “BAY 11‐7082 Inhibits the NF‐κB and NLRP3 Inflammasome Pathways and Protects Against IMQ‐Induced Psoriasis,” Clinical Science 131, no. 6 (2017): 487–498, 10.1042/CS20160645. 28096316

[38] Y. Chen , P. Lian , Z. Peng , et al., “Alpha‐7 Nicotinic Acetylcholine Receptor Agonist Alleviates Psoriasis‐Like Inflammation Through Inhibition of the STAT3 and NF‐κB Signaling Pathway,” Cell Death Discovery 8, no. 1 (2022): 141, 10.1038/s41420-022-00943-4. PMC8964744 35351863

[39] H. Hou , J. Li , J. Wang , R. Hou , J. Li , and K. Zhang , “Abnormal Dermal Microvascular Endothelial Cells in Psoriatic Excessive Angiogenesis,” Microvascular Research 155 (2024): 104718. 10.1016/j.mvr.2024.104718 39019108

[40] M. S. Zeini , N.‐S. Haddadi , M. Shayan , et al., “Losartan Ointment Attenuates Imiquimod‐Induced Psoriasis‐Like Inflammation,” International Immunopharmacology 100 (2021): 108160. 10.1016/j.intimp.2021.108160 34583123

[41] V. Koivukangas , M. Kallionen , J. Karvonen , et al., “Increased Collagen Synthesis in Psoriasis In Vivo,” Archives of Dermatological Research 287 (1995): 171–175, 10.1007/BF01262327. 7763089

[42] I. A. Rather , V. K. Bajpai , Y. S. Huh , et al., “Probiotic Lactobacillus sakei proBio‐65 Extract Ameliorates the Severity of Imiquimod Induced Psoriasis‐Like Skin Inflammation in a Mouse Model,” Frontiers in Microbiology 9 (2018): 1021, 10.3389/fmicb.2018.01021. PMC5968580 29867905

[43] R. Chen , Y.‐Y. Zhai , L. Sun , et al., “Alantolactone‐Loaded Chitosan/Hyaluronic Acid Nanoparticles Suppress Psoriasis by Deactivating STAT3 Pathway and Restricting Immune Cell Recruitment,” Asian Journal of Pharmaceutical Sciences 17, no. 2 (2022): 268–283, 10.1016/j.ajps.2022.02.003. PMC9091614 35582636

[44] X. Chen , G. Deng , K. Chen , Y. Chen , W. Ye , and P. Sun , “Targeting the NLRP3 Inflammasome in Psoriasis,” International Journal of Dermatology. Jul 63, no. 7 (2024): 844–851, 10.1111/ijd.17073. 38345734

[45] Y. Matsushima , S. T. Hwang , and S. I. Simon , “Mechanobiology of Neutrophil Inflammasome Signaling in Psoriasis,” Current Opinion in Immunology 97 (2025): 102680, 10.1016/j.coi.2025.102680. 41108802

