# Supplementary material for: Loganin Modulates Inflammatory Signalling and Epidermal Alterations in an Imiquimod‐Induced Psoriasis Mouse Model
Source: J Biochem Mol Toxicol. 2026 Nov 10;40(11):e71178. doi: 10.1002/jbt.71178 (PMC13749415; doi:10.1002/jbt.71178)
Supplement: Supplementary file 1 [file JBT-40-e71178-s001.docx]

**Supplementary Data**

**Supp. Figure 1.** Body weight change graph for animals across all groups during the experiment. Data are mean ± SEM (n = 7). *^⁎⁎⁎⁎^*p < 0.0001 versus the IMQ group. (Cont: Control; IMQ: Imiquimod; LOG: Loganin; SAL+VAS: Saline + Vaseline)

**Supp. Figure 2.** MT staining microscopic images of collagen fibres. A. Collagen fibres in the dermis of the dorsal skin sections (blue-stained areas), **B**. Collagen fibres in the dermis of the right ear skin sections (blue-stained areas), **C.** Collagen density graph in the dermis. For collagen fibre density analysis, five non-overlapping microscopic fields from different tissue regions were evaluated per mouse and averaged to obtain a single animal-level value prior to statistical analysis. Data are presented as mean ± SEM (n = 7 mice/group). Data are presented as mean ± SEM (n = 7 mice/group, *p* > 0.05). (MT: Masson’s trichrome, scale bar: 100 μm and 50 μm; magnification: 20x and 40x). (Cont: Control; IMQ: Imiquimod; LOG: Loganin; SAL+VAS: Saline + Vaseline)

**Supp. Figure 3.** Results of stereological analysis of epidermis and dermis volume density changes in dorsal skin sections. A. Total number of spots in the epidermis per corresponding area, B. Total epidermis volume (mm³), C. Ratio of epidermis volume/epidermis + dermis volume, D. Total number of spots in the dermis per corresponding area, E. Total dermis volume (mm³), F. Ratio of dermis volume/epidermis + dermis volume. Data are mean ± SEM (***p*<0.01; ****p*<0.001). (Cont: Control; IMQ: Imiquimod; LOG: Loganin; SAL+VAS: Saline + Vaseline)

| **Gene** | **Forward primer (5′–3′)** | **Reverse primer (5′–3′)** | **GenBank accession no.** |
| --- | --- | --- | --- |
| *Nlrp3* | TCACAACTCGCCCAAGGAGGAA | AAGAGACCACGGCAGAAGCTAG | NM_145827.4 |
| *ASC(Pycard)* | CGGAAAGTGGAATCCTTGCAGG | AGCAGTGAGGTCAGGCTTGGAA | NM_023258.5 |
| *Casp1* | GGCACATTTCCAGGACTGACTG | GCAAGACGTGTACGAGTGGTTG | NM_009807.3 |
| *Il1b* | TGGACCTTCCAGGATGAGGACA | GTTCATCTCGGAGCCTGTAGTG | NM_008361.4 |
| *Gsdmd* | **ATCCTGGCATTCCGAGTGG** | **CTCTGGCCCACTGCTTTTCT** | **NM_026960** |
| *Tnf* | **GCGACGTGGAACTGGCAGAAG** | **GCCACAAGCAGGAATGAGAAGAGG** | **NM_0136** |
| *Il17a* | CTCCAGAAGGCCCTCAGACTAC | GGGTCTTCATTGCGGTGG | NM_010552.3 |
| *Actb* | GGCTGTATTCCCCTCCATCG | CCAGTTGGTAACAATGCCATGT | NM_007393.5 |

**Supplementary Table S1.** Primer sequences and corresponding GenBank accession numbers used for quantitative real-time PCR (qRT-PCR) analysis. Forward and reverse primer sequences are presented in the 5′–3′ direction. Gene symbols are reported according to standard mouse gene nomenclature.
